# The epidemic of HIV and syphilis and the correlation with substance abuse among men who have sex with men in China: A systematic review and meta-analysis

**DOI:** 10.3389/fpubh.2023.1082637

**Published:** 2023-02-17

**Authors:** Tian Zhao, Guohong Chen, Chengqing Sun, Xiangdong Gong, Huiyong Li, Gengfeng Fu

**Affiliations:** ^1^School of Public Health, Nanjing Medical University, Nanjing, China; ^2^Jiangsu Provincial Center for Disease Control and Prevention, Institute for STI and HIV Control and Prevention, Nanjing, China; ^3^Department of Epidemiology, National Center for STD Control, Nanjing, China; ^4^Department of STI and HIV Control and Prevention, Jincheng Center for Disease Control and Prevention, Jincheng, China

**Keywords:** HIV, syphilis, substance abuse, men who have sex with men (MSM), correlation

## Abstract

**Background:**

In China, the HIV/AIDS epidemic among men who have sex with men (MSM) has been expanding in recent years. Substance abuse in MSM was not well studied as the independent risk factor for HIV and syphilis infection and other sexually transmitted diseases. The present review aimed to determine the correlation between HIV/Syphilis infections and substance abuse and other sexual risk behaviors among MSM.

**Methods:**

We conducted a comprehensive search of PubMed, Web of Science, Embase, Scopus, Chinese National Knowledge Infrastructure, Chinese Wanfang Data, and VIP Chinese Journal Database for relevant articles of quantitative studies published between 2010 and May 31, 2022. Meta-analysis was performed using R software. Pooled estimated of the association-odds ratio, with 95% confidence intervals were calculated using random-effects models stratified by study design. Q statistics and I^2^ were used to measure the heterogeneity.

**Results:**

Our meta-analysis included 61,719 Chinese MSM from 52 eligible studies. The pooled HIV prevalence rate among substance-abusing MSM was 10.0% (95% CI = 0.08–0.13). Substance abusers were more likely to have a higher prevalence of HIV (OR = 1.59) and syphilis (OR = 1.48) infections than non-substance abusers. Substance abusers were also more likely to seek sexual partners through the internet or social media applications (OR = 1.63), engage in unprotected anal intercourse (UAI) (OR = 1.69), group sex (OR = 2.78), and engage in commercial intercourse (OR = 2.04) compared to non-users. Regarding testing behaviors, substance abusers had a higher proportion of HIV or STI testing in their lifetime (OR = 1.70) compared with non-substance abusers (*p* < 0.05). They were also more likely to have had more sexual partners (≥2; OR = 2.31) and more likely to have consumed alcohol (OR = 1.49) in the past 6 months.

**Conclusions:**

Our study shows the correlation between substance abuse and HIV/Syphilis infection. Eliminating disparities in HIV/Syphilis infection among substance abusing men who have sex with men (MSM) can be achieved if the Chinese government and public health sectors could provide targeted knowledge popularization and diagnosis interventions among high-risk populations.

## 1. Introduction

In China, the HIV/AIDS epidemic is increasing among men who have sex with men (MSM). By the end of 2020, the estimated number of people living with HIV was 2.1 million worldwide, with homosexual transmission accounting for 45.0% of reported HIV/AIDS cases (1). The HIV epidemic has exacted a severe toll on MSM, with HIV infections from 9.1% in 2009 to 23.3% in 2020 (2). In addition, the prevalence of HIV and syphilis co-infection has increased from 8% (Mexico, 2010–2018) to 25% (Turkey, 2013) (3, 4). Previous studies have found that syphilis infections could potentially facilitate HIV transmission and increase the likelihood of acquiring HIV (5). The increasing HIV/AIDS epidemic among MSM is undoubtedly a major public health problem. Therefore, clarifying the risk factors associated with HIV acquisition among Chinese MSM is urgently needed to HIV prevention and intervention strategies.

Similarly, the global rates of substance abuse among men who have sex with men have increased dramatically. Evidence from European countries and China suggests that methamphetamine and ecstasy are the most popular drugs used by MSM in the past years (4–6). A recent study among 3,588 number of participants that explored drug use problems found that the use of nitrite inhalers have overtaken methamphetamine as the most popular substance abused among MSM (7). Chinese MSM has increasingly purchased poppers, and the proportion of poppers used among MSM was 24% in 2020 (8, 9). The recreational use of drugs like rush poppers are more likely to be associated with sex and may thus be linked to the transmission of HIV and other sexually transmitted infections (STI).

On the one hand, drugs like meth/amphetamine, are often used to increase sexual desire and facilitate sexual experimentation (10). Rush popper may reduce pain by dilating capillaries and relaxing anal sphincters, increasing sexual confidence. However, substance abuse has both physiological and psychological effects on users. It may facilitate risky sexual practices, such as unprotected anal intercourse (UAI), thereby potentially increasing the risk of HIV and STI transmission (11–16), substance abuse and UAI are co-occurring risk behaviors that may contribute to the HIV infection rates among MSM.

Over the past years, research studies have explored the association between substance abuse and other risk behaviors that might contribute to HIV infection in the MSM population (6, 15–20). However, the results of these studies have been inconsistent on the significance of this association, previous meta-analyses on substance abuse among MSM or HIV infection failed to provide evidence of underlying differences in HIV-related risk behaviors and associated factors between substance abusers and non-users (21, 22). Still, more evidence is needed. This quantitative review aimed to explore the risk factors associated with higher HIV/Syphilis infection rates among MSM substance abusers compared to the general MSM population. We focused on the sexual risk behaviors between substance abusers and non-users. Furthermore, understanding these issues could further facilitate effective intervention and prevention strategies among Chinese MSM.

## 2. Methods

### 2.1. Literature search strategy

This meta-analysis adhered to the PRISMA guidelines. We conducted a comprehensive search of PubMed, Web of Science, Embase, Scopus, Chinese National Knowledge Infrastructure, Chinese Wanfang Data, and VIP Chinese Journal Database for relevant studies with quantitative outcomes associated with HIV/Syphilis infection among men who have sex with men (MSM). And these articles published in English or Chinese between 2010 and May 31, 2022. Meta-analyses were performed to compare HIV and syphilis risks between substance abusers and non-users across studies.

An appropriate combination of keywords and the MeSH subject headings for the search, including (1) “men who have sex with men” OR “MSM” OR “gay man” OR “male homosexual” AND (2) (“HIV infection”) OR “HIV”AND (3) “Club drug use” OR “Recreational drug use” OR “Illicit drug use” OR “Substance abuse.” AND (4) “China.” We also reviewed the bibliographies of included citations to identify references for consideration.

### 2.2. Inclusion and exclusion criteria

The inclusion criteria were as follows: (1) cross-sectional or longitudinal study design; (2) MSM as a target population; (3) the main outcome focus was HIV prevalence rate or HIV incidence; (4) reported the proportion of substance abuse among MSM in China; (5) were published in English or Chinese and conducted in China; (6) Sample sizes >50; (7) was a master or doctoral thesis which satisfied the above requirements.

Studies were excluded for the following reasons: (1) the study population was not MSM; (2) it exclusively focused on MSM living with HIV or substance abusers; (3) the studies without relevant quantitative data; (4) the study was a systematic review. The most comprehensive studies were included in the meta-analysis.

### 2.3. Study screening and data extraction

We imported the literature into Zotero to build a library, eliminated duplicates, and two authors (GH and ZT) screened the titles and abstracts for eligibility. According to the inclusion and exclusion criteria, two authors (CQ and ZT) conducted the full text screening. Discrepancies were discussed until agreement was reached, with an arbitrator (Gengfeng Fu) for unresolved disagreement.

Two authors (GH and ZT) used a standardized form for data extraction, including the following information: source (the primary author, publication year), study periods, sample size, study setting (city, state), recruitment strategy, survey methods, and recall window; types and proportion of substances abused; population characteristics (e.g., sexual risk behaviors including unprotected anal intercourse, the number of male sexual partners, engaging in commercial sex or group sex); the proportion of reporting HIV-positive and syphilis-positive. All abstracted data were used to calculate prevalence rate ratios and relative risk.

### 2.4. Quality assessment

We used the Quality Assessment Checklist (QATSO score) (23) for quality assessment. This checklist is a validated quality assessment tool for HIV prevalence/risk behaviors among MSM (the checklist is provided in Supplementary material). The scoring system was based on five items: (1) Whether the sampling method is representative of the population of the study; (2) the measurement of the HIV objective (if the article is focusing only on risk behavior among MSM); (3) Whether response rate is reported in studies; (4) Whether confounding bias is controlled (such as stratification/matching/restriction/adjustment) when analyzing the associations; (5) Whether privacy or the sensitive nature of HIV is considered when conducting the survey. According to these quality items, the scores are 1, 0, and NA, representing “yes,” “no,” and “not applicable,” respectively. All scores were categorized into poor (0%−32%), satisfactory (33%−66%), and good (67%−100%) groups. Quality assessment scores are provided in Supplementary material.

### 2.5. Statistical analysis

Meta-analysis was performed using R software and was stratified by study design. We used a random -effects model to aggregate every outcome and estimated the pooled association between substance abuse and HIV infection and their 95% CI. Q statistics defined the heterogeneity between studies. Based on the I^2^ classification suggested by Higgins and Thompson, we used the cut-off of 25, 50, and 75% to define as low, medium, and high levels of heterogeneity, respectively. If significant homogeneity was detected (I^2^ > 50%), random effect models were employed to calculate the pooled effect rates and OR, otherwise, fixed effects models were employed.

Another subgroup analysis was performed to explore the sources of heterogeneity in these studies, such as sampling size, study periods, and study setting. Egger's regression test and the funnel plot evaluated the possible publication bias and the effect of small sample sizes. Finally, the sensitivity analysis was performed to explore the possible impact of abnormal or outlier data. We used R software (version 4.2.0) to conduct all statistical analyses.

## 3. Results

### 3.1. Overview of studies

**Figure 1:**
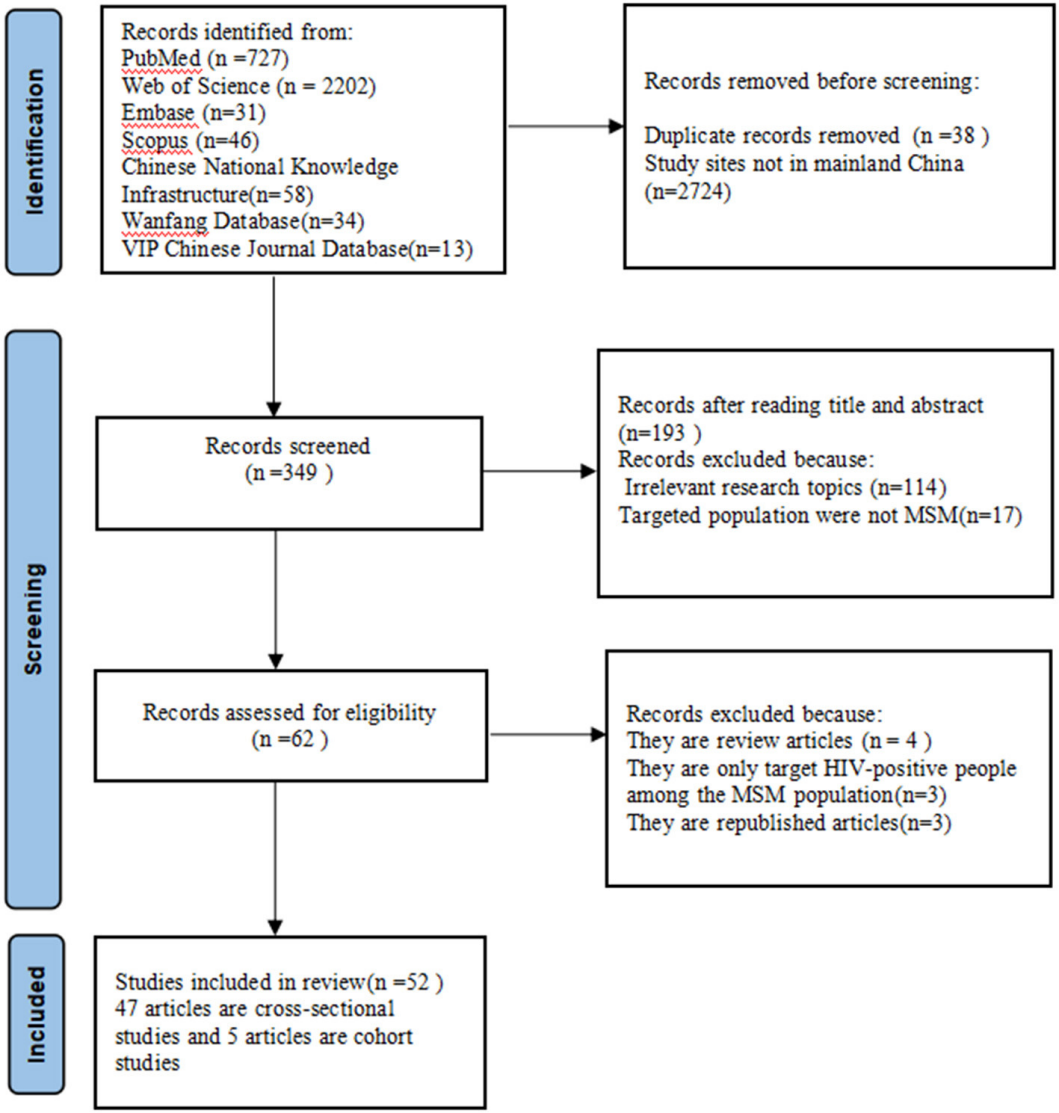
Flowchart showing the selection of study subjects.

We identified 3,111 unique articles, 3,049 of which did not meet the inclusion criteria, and 62 articles progressed to full-text screening. Eventually, 52 eligible articles (including 61,719 MSM) were selected in this meta-analysis (7–9, 18–20, 24–69). The flow of the review process is shown in Figure 1.

### 3.2. Basic characteristics of the selected studies

All included papers contained 47 cross-sectional studies and 5 cohort studies. A total of 61,719 MSM was included across the included studies, with a maximum sample size of 6,710, a minimum sample size of 139, and 50 studies with more than 200 participants. The study location involved 24 cities and 11 provinces, and the sampling methods included peer referrals, snowball method, internet recruitment, voluntary counseling test, and venue-based mobilization. Five studies used voluntary counseling test (VCT) method to recruit MSM population, and seven studies used snowball sampling method to recruit participants. Other forty studies used more than one sampling methods to recruit participants. The survey methods were based on self-administrated questionnaires and a face-to-face interview. The characteristics of the studies selected for this review are summarized in Table 1.

**Table 1:** Study characteristics of 52 samples included in meta-analysis.

**References**	**Type of study**	**Study location**	**Study periods**	**Recruitment methods**	**Survey methods**	**Eligibility of subjects**	**Sample size**	**Recall window (months)**	**Types of substances abuse**	**Substance abuse rate**	**HIV prevalence rate of substance abusers**
Liu et al. (7)	Cross-sectional	Shenzhen	2018.6–2018.9	VCT	Self-administrated questionnaire	Self-reported having anal or oral intercourse with other men in the past 6 months	455	6	Methamphetamine, Ketamine, MDMA	14.90	NA
Xi (46)	Cross-sectional	Hangzhou	2009.6–2010.6	Snowball sampling	Self-administrated questionnaire	>18 years; self-reported having anal or oral intercourse with other men in the past 6 months	530	6	MDMA, Ketamine	3.77	5.00
Hu (33)	Cross-sectional	Coastal cities in eastern China	2013.4–2013.10	Website advertisement; community outreach; peer referrals; VCT	Self-administrated questionnaire; individual interview	≥18 years; self-reported having anal intercourse with other men in the 6 months; local residence; reporting the use of synthetic drugs	447	6	Methamphetamine, Magu, Rush Poppers, Cannabis, Sildenafil	51.50	NA
Li et al. (37)	Cross-sectional	Beijing	2012.6–2012.12	Website advertisements; community outreach; peer referrals; VCT	Self-administrated questionnaire	≥18 years, having sex with men during the past 3 months, can provide blood samples to test for HIV and willing to provide written informed consent	400	6	Nitrite inhalants	47.30	9.00
He et al. (32)	Cross-sectional	Guangzhou	2013.11–2014.6	VCT	Self-administrated questionnaire	Self-reported having anal intercourse with other men in the 2 years	1,046	6	Rush poppers	1.24	NA
Liu et al. (40)	Cross-sectional	Nanjing	2013.4–2013.6	Category snowball sampling	Face-to-face interview	≥18 years; self-reported having anal intercourse with other men in the past 6 months; living in Nanjing for more than 3 months	566	12	Hallucinogens	2.12	NA
Wang et al. (45)	Cross-sectional	Beijing	2012.12–2013.7	Outreaching in gay venues; web advertisement and peer referral	Self-administrated questionnaire	Chinese men age 18–60 years old, living in Beijing, having anal intercourse with at least one man in the last 3 months	576	3	Nitrite inhalants	28.60	NA
Chen et al. (25)	Cross-sectional	Changsha	2009.8–2009.11	Snowball sampling; internet advertisements; venue-based mobilization	Self-administrated questionnaire	≥16 years; self-report sexual behaviors with a male partner at least once in their lifetime; understood and were willing to participate and sign informed consent	826	6	ketamine, ecstasy/MDMA, GHB, cocaine, and methamphetamine	21.40	18.64
Cai et al. (27)	Cross-sectional	Shenzhen	2010–2014	VCT	Self-administrated questionnaire	≥18 years; self-reported having sex with other men	3,318	24	Heroin, Cocaine, Opium, Ketamine, MDMA, Methamphetamine	4.65	NA
Shi et al. (42)	Cross-sectional	Wuhan	2013.5–2013.11	VCT; peer referrals; website advertisement; community outreach activities	Self-administrated questionnaire	< 25 years; self-reported having anal or oral intercourse with other men in the past 6 months	397	6	NA	22.90	12.09
Wang (44)	Cross-sectional	Kunming	2015	Snowball sampling^;^website advertisement; Purpose Sampling	Self-administrated questionnaire; In-depth personal interviews	≥18 years; self-reported having oral or anal intercourse with other men in; living and working in Kunming for more than 3 months; high school students or non-students	635	6	Rush poppers, Methamphetamine	35.11	NA
Chen et al. (28)	Cross-sectional	Guangdong province	2014.3–2014.8	Homosexual website	Self-administrated questionnaire;	>16 years; self-reported having anal intercourse with other men; Students from universities in Guangdong Province	825	3	Rush poppers	14.80	NA
Li et al. (64)	Cross-sectional	Beijing	2013.4–2013.12	Website advertisement; community outreach; peer referrals; venue-based mobilization	Self-administrated questionnaire	≥18 years; self-reported having oral or anal intercourse with other men in; reporting the use of synthetic drugs in the past 12 months; living and working in Beijing	1,206	3	Rush poppers	23.55	NA
Yang et al. (50)	Cross-sectional	Hunan Province	2012.7–2013.1	Respondent driven sampling;	Self-administrated questionnaire	Males aged >16 years; self-identified as an MB; ability to provide written and verbal consent	205	3	Rush popper, methamphetamine, ecstasy, ketamine, marijuana, morphine, heroin and cocaine	39.02	NA
Chen et al. (28)	Cross-sectional	Beijing	2013–2016	Website advertisement; peer referral; short message services and community outreach	Self-administrated questionnaire	≥18 years; having sex with men in the past 12 months; living in Beijing during the study period; willing and able to provide written informed consent	3,588	3	Rush poppers, methamphetamine, cannabis or marijuana, MDMA, ketamine, Magu and heroin	27.50	16.01
Jiang (36)	Cross-sectional	Chongqing	2015.12–2016.4	Snowball sampling; occasional Sampling	Self-administrated questionnaire	>16 years; self-reported having anal or oral intercourse with other men in the past year; self-reported using new drugs in the last year	140	12	Methamphetamine, Magu, Capsule Zero, Amphetamine, Ketamine, Rush poppers	100%	NA
Zhu et al. (56)	Cross-sectional	Nanjing	2014.4–2014.6; 2014.12–2014.12; 2015.4–2015.6	Internet recruitment ads; VCT; community Organization Mobilization	Self-administrated questionnaire	>16 years; self-reported having anal or oral intercourse with other men in the last year	1,721	6	Rush poppers	19.29	16.87
Xu et al. (47)	Cross-sectional	Nanjing	2014.11–2015.2; 2015.4–2015.7	Social networks; venue-based mobilization; VCT; community Organization Mobilization	Self-administrated questionnaire	>16 years; self-reported having anal or oral intercourse with other men in the last year; living in Nanjing	1,040	6	Rush poppers	23.65	18.70
Yang et al. (9)	Cross-sectional	Luoyang	2016.5–2016.10	Snowball sampling	Self-administrated questionnaire	Men over 16 years old; self-reported having anal intercourse with other men	1,010	24	Methamphetamine, Ketamine, MDMA, Magu, Rush poppers, Capsule Zero	79.40	NA
Jia et al. (35)	Cross-sectional	Xian	2016	Snowball sampling	Self-administrated questionnaire	Self-reported having anal or oral intercourse with other men in the past 3 months	214	3	Methamphetamine, Ketamine, Magu, Rush poppers, Capsule Zero	39.70	9.41
Zhang et al. (54)	Cross-sectional	Beijing, Nanning	2013.3–2014.3	Short message service (SMS); community outreach; web advertisement and peer referral interventions	Self-administrated questionnaire	Male, at least 18 years old, self-reported sex with men in the last 12 months, currently living in Beijing, and provision of written informed consent	500	3	Rush poppers, methamphetamine, marijuana, ecstasy, ketamine, Magu.	38.60	6.22
Wang et al. (20)	Cross-sectional	Beijing	2013.4–2014.4	Online advertising and peer referral	Face-to-face interview	≥18 years; self-reported had anal intercourse at least once with another male in the past 12 months; and currently living in Beijing (north China) or Nanning (south China	510	6	Nitrite inhalants	29.62	14.57
Shan et al. (41)	Cross-sectional	Tianjin	2016.4–2016.12	Snowball sampling;website advertisement; community outreach; peer referrals; VCT; venue-based mobilization	Self-administrated questionnaire	≥18 years; self-reported having oral or anal intercourse with other men in the past 6 months; reporting the use of synthetic drugs in the past 6 months	302	6	Rush poppers, Capsule Zero	100%	NA
Duan et al. (30)	Cross-sectional	Shenzhen	2015.3–2015.12	Time-location-sampling (TLS); the respondent driven sampling (RDS); snowball sampling	Self-administrated questionnaire	Being biologically male; having had sexual contact (oral or anal sex) with other males in Shenzhen during the past 6 months before the survey	1,935	6	Rush poppers, methamphetamine, ketamine, LSD, PCP, marijuana, cocaine, ecstasy (amphetamine, MDMA), cough syrup (codeine phosphate), heroin, Magu	12.70	18.29
Zhao et al. (24)	Cross-sectional	Nation-wide	2014.9–2014.10	Three gay web portals: Northern China, Southern China, and Eastern China	Self-administrated questionnaire	Being born as male; ≥16 years; had ever engaged in anal sex with another man; willing to provide their cell phone number (for other follow-up purposes) and agreed to an informed consent	1,424	12	Rush poppers, crystal meth and ecstasy	24.72	4.83
Han (31)	Cross-sectional	Tianjin	2015.7–2017.9	Snowball sampling; Simple random Sampling	Self-administrated questionnaire	>16 years; self-reported having anal intercourse with other men in the past 3 months; HIV negative or self-reported unknown	222	3	Methamphetamine, Rush poppers, Capsule Zero, Ketamine, Magu	54.50	5.78
Lu (26)	Cross-sectional	Qingdao	2017.3–2017.8	Snowball sampling	Face-to-face interview	>18 years; self-reported having anal or oral intercourse with other men in the past 6 months; local residence	602	6	Methamphetamine, Rush poppers, Capsule Zero, MDMA, Ketamine, Magu	50.20	0.33
Zhou et al. (57)	Cross-sectional	Nantong	2018.4–2018.8	Snowball sampling; social networks; venue-based mobilization; VCT	Self-administrated questionnaire	Men self-reported having anal or oral intercourse with other men in the last year	300	6	Rush poppers, Capsule Zero, MDMA	64.00	4.90
Li (39)	Cross-sectional	Haerbin	2016.4–2017.3	Snowball sampling; Internet recruitment ads; VCT; community organization Mobilization; social networks	Self-administrated questionnaire	>18 years; self-reported having anal or oral intercourse with other men	6,710	12	Rush poppers, Amphetamine, Ketamine, MDMA, Cannabis, Capsule Zero	23.10	11.61
Zhang (53)	Cross-sectional	Beijing, Tianjin, Shijiazhuang	2018.11–2019.1	VCT; peer referrals; social networks	Self-administrated questionnaire	Men over 18 years old; self-reported having anal intercourse with other men in the past 6 months; full-time enrolled college students; Working or living in Beijing or Tianjin or Shijiazhuang during the survey	511	6	Rush poppers, Capsule Zero	55.00	NA
He et al. (68)	Cross-sectional	Hangzhou	2015.8–2016.4	Snowball sampling	Face-to-face interview	≥16 years; had had anal sex with men in the last 3 months before the study; had previous HIV test results that were negative or unknown; and were willing to provide informed consent to participate in the study	555	3	Methamphetamine, ketamine, ecstasy, and rush poppers,	18.20	15.80
Lan et al. (69)	Cross-sectional	Guangxi province (Guigang, Guilin, Hechi, Hezhou, Liuzhou, Nanning, Wuzhou, and Yulin)	2013–2015	Advertisements; peer recruiters; peer referral	Face-to-face interview	≥18 years; self-report same-gender sex in the past 6 months; currently living in the selected cities and willing to provide written informed consent	5,658	6	NA	0.76	23.26
Duan (8)	Cross-sectional	Jinan, Qingdao, Yantai	2016.4–2016.7	Snowball sampling; social networks; venue-based mobilization	Self-administrated questionnaire	>16 years; self-reported having anal intercourse with other men in the past year; living in Jinan, Qingdao or Yantai during the survey	1,306	6	Methamphetamine, MDMA, Ketamine, Rush Poppers, Capsule Zero	28.60	6.95
Xu et al. (48)	Cross-sectional	Nanjing	2016.4–2016.7	Internet recruitment ads; peer referral	Self-administrated questionnaire	>16 years; self-reported having anal or oral intercourse with other men in the last year; living in Nanjing	876	6	Rush Poppers, Capsule Zero, Methamphetamine, MDMA, Codeine phosphate, Magu, Ketamine, Amphetamine	29.60	13.90
Dai et al. (29)	Cross-sectional	Sichuan province (Deyang, Yibin, and Xichang)	2016.6–2016.9	Community-based organizations	Self-administrated questionnaire	≥18 years; reported sexual experience with men; currently living in the selected cities and willing to provide informed consent	1,122	12	Rush Poppers, crystal meth and capsule zero	27.70	21.54
Zhou et al. (55)	Cross-sectional	Tianjin	2012.4–2012.8	Community recruitment	Self-administrated questionnaire	≥18 years; self-reported having sex with other men in the past 6 months; HIV infection status is negative or unknown	500	6	Rush Poppers	36.60	6.22
Wang et al. (66)	Cross-sectional	Guangzhou, Shenzhen, Wuxi	2017.1–2017.8	Snowball sampling; VCT; website advertise	Self-administrated questionnaire	>18 years; self-reported having anal intercourse with other men or have at least 2 male sexual partners in the past 6 months or Having a sexually transmitted disease	603	6	Methamphetamine, Rush Poppers, Sildenafil	25.50	8.44
Li et al. (65)	Cross-sectional	Chongqing	2018.6–2019.6	Snowball sampling;website advertisement; peer referrals,	Self-administrated questionnaire	Men age 18–65 years old; being born as male or transgender women who have sex with men; HIV-negative	139	6	Rush Poppers, Methamphetamine, Ketamine, Capsule Zero, Diazepam, Codeine phosphate	34.53	NA
Huang et al. (34)	Cross-sectional studies	Jinan Qingdao	2016.3–2016.6	Venue-based mobilization; Online recruitment	Self-administrated questionnaire	Self-reported having oral or anal intercourse with other men in the past year	901	6	Rush Poppers	30.10	2.95
Guo et al. (63)	Cross-sectional	Beijing, Tianjin	2019.11–2019.11	Snowball sampling; peer referrals	Self-administrated questionnaire	Men age 15–30 years old in high school or graduation within one year; self-reported having oral or anal intercourse with other men in the last year	220	3	Rush Poppers, Sildenafil	13.80	NA
Yan et al. (49)	Cross-sectional	Guangzhou	2017.5–2017.11	VCT	Self-administrated questionnaire	≥18 years; self-reported having anal intercourse with other men; living in Guangzhou for more than 3 months	976	6	Rush Poppers	34.84	NA
Chen et al. (67)	Cross-sectional	Chongqing	2019.3–2020.2	Convenience sampling	Self-administrated questionnaire	≥18 years; self-reported having anal intercourse with other men in the past year and were willing to provide written informed consent	1,151	6	Rush Poppers	17.72	26.96
Li et al. (19)	Cross-sectional	Hebei, Shandong, Jiangsu, Zhejiang, Fujian and Guangdong Province	2019.7–2019.12	VCT; Intervention Outreach Activities	Self-administrated questionnaire	≥18 years; self-reported having sex with other men; primary sexual orientation is male	2,690	24	Rush Poppers, Capsule Zero, Capsule Zero, Amphetamine	32.20	NA
Mao et al. (18)	Cross-sectional	Seven large cities in China (Shenyang, Jinan, Zhengzhou, Shanghai, Nanjing, Changsha, and Kunming)	2012.6–2013.6	Advertisements on gay websites; community-based organizations; peer referrals	Self-administrated questionnaire	Be born male, ≥16 years, self-reported having anal/oral sex experiences with a male within the last year, and able to provide informed consent	4,496	6	Rush popper, ecstasy, methamphetamine, amphetamine, codeine and ketamine.	28.36	12.71
Yu et al. (51)	Cross-sectional	Tianjin	2018.1–2018.12	Snowball sampling; website advertise; social networks	Face-to-face interview	Being born as male; < 24years; had ever engaged in sex with another man in the past 6 months; junior college, undergraduate, and postgraduate school enrollment	826	6	Methamphetamine, Rush Poppers, Capsule Zero	44.92	6.74
Wang et al. (43)	Cross-sectional	Jinan; Qingdao; Yantai	2020.4–2020.7	VCT	Self-administrated questionnaire	>15 years; self-reported using synthetic drug use; self-reported having anal or oral intercourse with other men in the last year	965	6	Methamphetamine, Rush poppers, Capsule zero and G-spot fluid	100%	NA
Li et al. (38)	Cross-sectional	Jinan	2017–2020	Jianzhuo Li−2022	Cross-sectional	Jinan	1,700	6	Capsule zero, Amphetamine, Rush poppers, MDMA	27.35	5.48
Chu and Shang (61)	Cohort	Shenyang	2011.1–2012.12	Respondent driven sampling	Self-administrated questionnaire	>18 years; self-reported having anal or oral intercourse with other men in the past year; no history of HIV-positive testing	625	3	Rush poppers, Methamphetamine, Codeine phosphate, Sildenafil	29.76	5.9/100PY
Peng and Xu (62)	Cohort	Liaoning	2017.6–2018.12	Snowball sampling; respondent driven sampling; WeChat promotion	Self-administrated questionnaire	>16 years; male by birth sex; self-reported having anal or oral intercourse with other men in the past year; no history of HIV-positive testing	672	6	Hallucinogens	36.80	5.8/100PY
Li (58)	Cohort	Tianjin	2013.4–2018.9	Social networks; venue-based mobilization	Self-administrated questionnaire	>16 years; self-reported having anal or oral intercourse with other men in the last 6 months; no history of HIV-positive testing; live in Tianjin for more than 6 months; awareness and willingness to participate in VCT surveys every 6 months	2,380	6	Rush poppers	20.60	2.23/100PY
Ni (59)	Cohort	Zhejiang province	2018.4–2020.4	VCT; Web recruitment	Self-administrated questionnaire	>16 years; self-reported having sex with other men in the past year; no history of HIV-positive testing; live in the local area for at least 1 year; able to provide contact information	731	6	Rush poppers, Capsule zero	NA	1.19/100PY
Shan et al. (60)	Cohort	Shanghai, Tianjin	2016.6–2018.6	Outreach; network intervention; peer education	Self-administrated questionnaire	>18 years; self-reported having anal or oral intercourse with other men in the past year; self-reported using substance in the past year; no history of HIV-positive testing	554	6	Rush poppers, Methamphetamine, Ecstasy, Sildenafil	100%	2.73/100PY

### 3.3. Analysis of substance abuse

**Figure 2:**
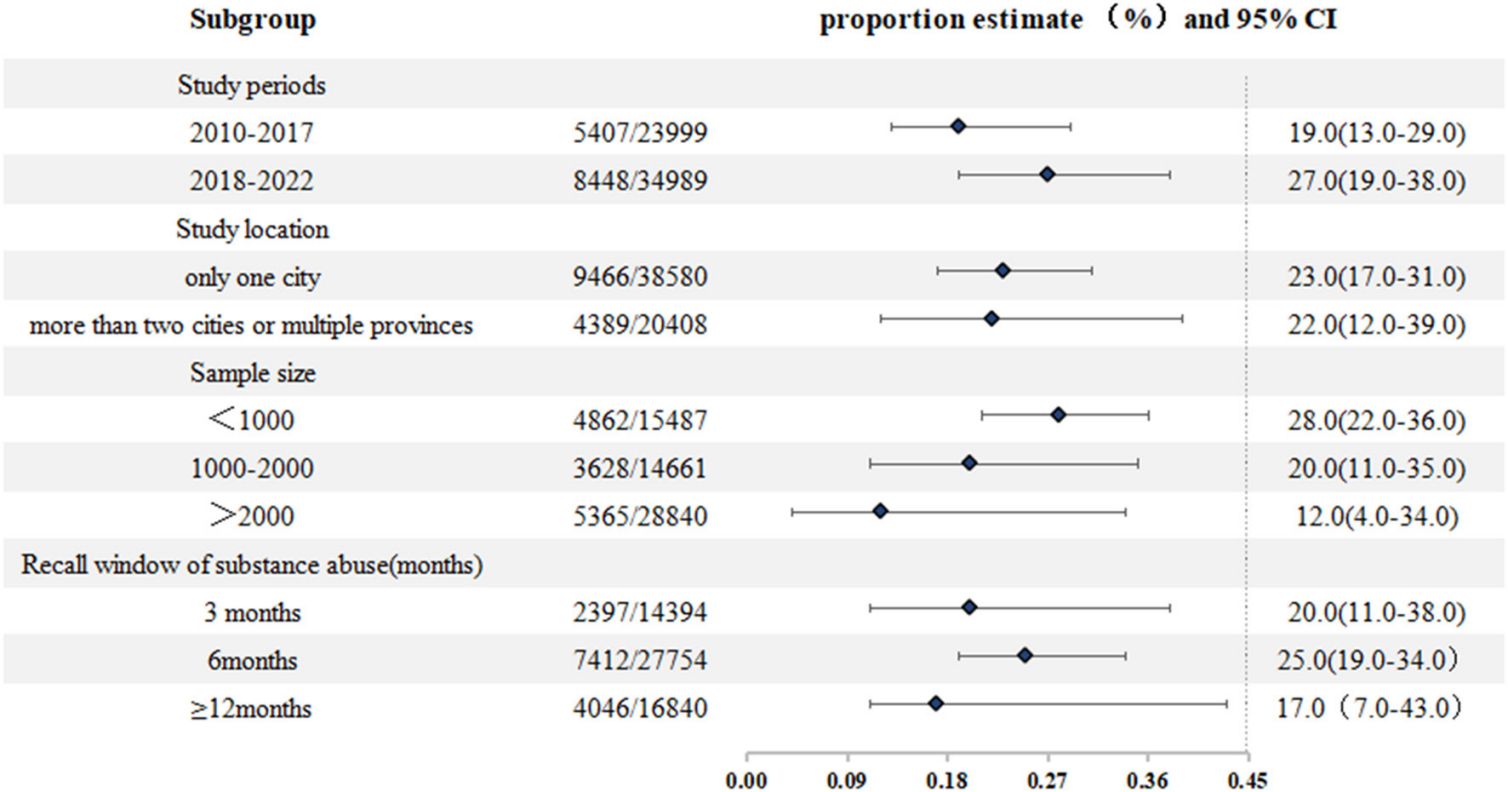
Subgroup analysis of substance abuse among Chinese MSM.

Among these selected articles, the pooled proportion of MSM who had ever used substance was 23% (95% CI: 0.17–0.30) (I^2^ = 99.3%; 95% CI: 0.93–0.94, *P* < 0.01), and in the past 6 months, a 25% (95% CI: 0.19–0.34) pooled proportion of Chinese MSM reported substance abuse. To examine the possible differences in the proportion of substance abusers, subgroup analyses were conducted by study period, study setting (city), sample size, and recall window (Figure 2).

Subgroup analysis indicated that the proportion of substance abuse varied at different study time intervals (19% from 2010 to 2017; 27% from 2018 to 2022). Whether the studies were conducted in a single city or in more than two cities, the differences in substance abuse rates were not significant (23% vs. 22%). The proportion of substance abuse was higher when the sample size was small: 28% for studies with 1,000 participants or fewer, 20% for 1,000–2,000 participants, and 12% for over 2,000 participants. Moreover, we also analyzed 26 studies conducted in 11 cities with strong comprehensive strength and the substance abuse rate in these cities (Beijing, Tianjin, Nanjing, Guangzhou, Shenzhen, Hangzhou, Xian, Wuhan, Changsha, Qingdao, and Chongqing) was 20% (95% CI: 0.14–0.28) (7, 20, 26, 31, 32, 35, 40, 42, 45–49, 51, 52, 55, 56, 58, 64, 65). Finally, the proportion of substance abuse was different when studies with a recall window of 12 months (17%), 6 months (25%), and 3 months (20%) (Table 2).

**Table 2:** Stratified meta-analyses of the proportion of substance abuse among MSM in China.

**Category**	**Subgroup**	**Number of studies**	**Proportion estimate (%) and 95% CI**	**Heterogeneity**	
				**I** ^2^ **/%**	***P*** **-value**
Study period	2010–2017	24	19.0 (13.0–29.0)	99	< 0.01
	2018–2022	23	27.0 (19.0–38.0)	99	< 0.01
Study location	Only one city	34	23.0 (17.0–31.0)	99	< 0.01
	More than two cities or multiple provinces	13	22.0 (12.0–39.0)	99	< 0.01
	11 cities with strong comprehensive strengths	26	20.0 (14.0–28.0)	99	< 0.01
Sample size	< 1,000	29	28.0 (21.0–36.0)	98	< 0.01
	1,000–2,000	11	20.0 (11.0–35.0)	99	< 0.01
	>2,000	7	12.0 (4.0–34.0)	99	< 0.01
Recall window of substance abuse (months)	3 months	12	20.0 (11.0–38.0)	99	< 0.01
	6 months	28	25.0 (19.0–34.0)	99	< 0.01
≥12 months	7	17.0 (7.0–43.0)	99	< 0.01

### 3.4. Prevalence of HIV and syphilis

Of the 47 cross-sectional studies in the meta-analysis, 12 did not report HIV prevalence rates, and the other 35 used HIV ELISA testing to confirm HIV infection status. So we used data from the 35 studies to compare HIV prevalence rates among substances abusers and non-users (8, 18, 20, 24–27, 29, 31–44, 46–48, 51, 52, 55–57, 64, 66, 68, 69).

Twenty-eight studies contributed findings to aggregate the HIV prevalence of substance abusers and non-users among MSM (10.0% vs. 7.0%), the pooled prevalence rate of HIV among substance abusers was 10.0% (95% CI: 0.08–0.13) (I^2^ = 88%; 95% CI: 0.88–0.91, *P* < 0.01). MSM who reported ever using substances were 1.59 times more likely to be HIV positive than non-users [Pooled OR = 1.59 (95% CI: 1.33–1.89); I^2^ = 59%; *P* < 0.01] (Table 3).

**Table 3:** Stratified meta-analyses of the proportion of HIV prevalence among MSM in China.

**Category**	**Subgroup**	**Number of studies**	**Proportion estimate and 95% CI**	**Heterogeneity**	
				**I** ^2^ **/%**	***P*** **-value**
Study population	Substance abusers	28	0.10 (0.08–0.13)	88	< 0.01
	Non-users	28	0.07 (0.06–0.09)	89	< 0.01
Study year	2011–2017	18	0.11 (0.08–0.13)	93	< 0.01
	2018–2022	17	0.07 (0.05–0.09)	95	< 0.01
Survey method	Self-administrated questionnaire	29	0.09 (0.07–0.11)	95	< 0.01
	Face-to-face interview	6	0.04 (0.01–0.13)	94	< 0.01
Sample size	< 1,000	22	0.08 (0.06–0.10)	94	< 0.01
	1,000–2,000	9	0.09 (0.06–0.13)	96	< 0.01
	>2,000	4	0.10 (0.08–0.12)	94	< 0.01
Study location	Only one city	26	0.09 (0.07–0.11)	94	< 0.01
	>2 cities	9	0.07 (0.05–0.09)	94	< 0.01

**Figure 3:**
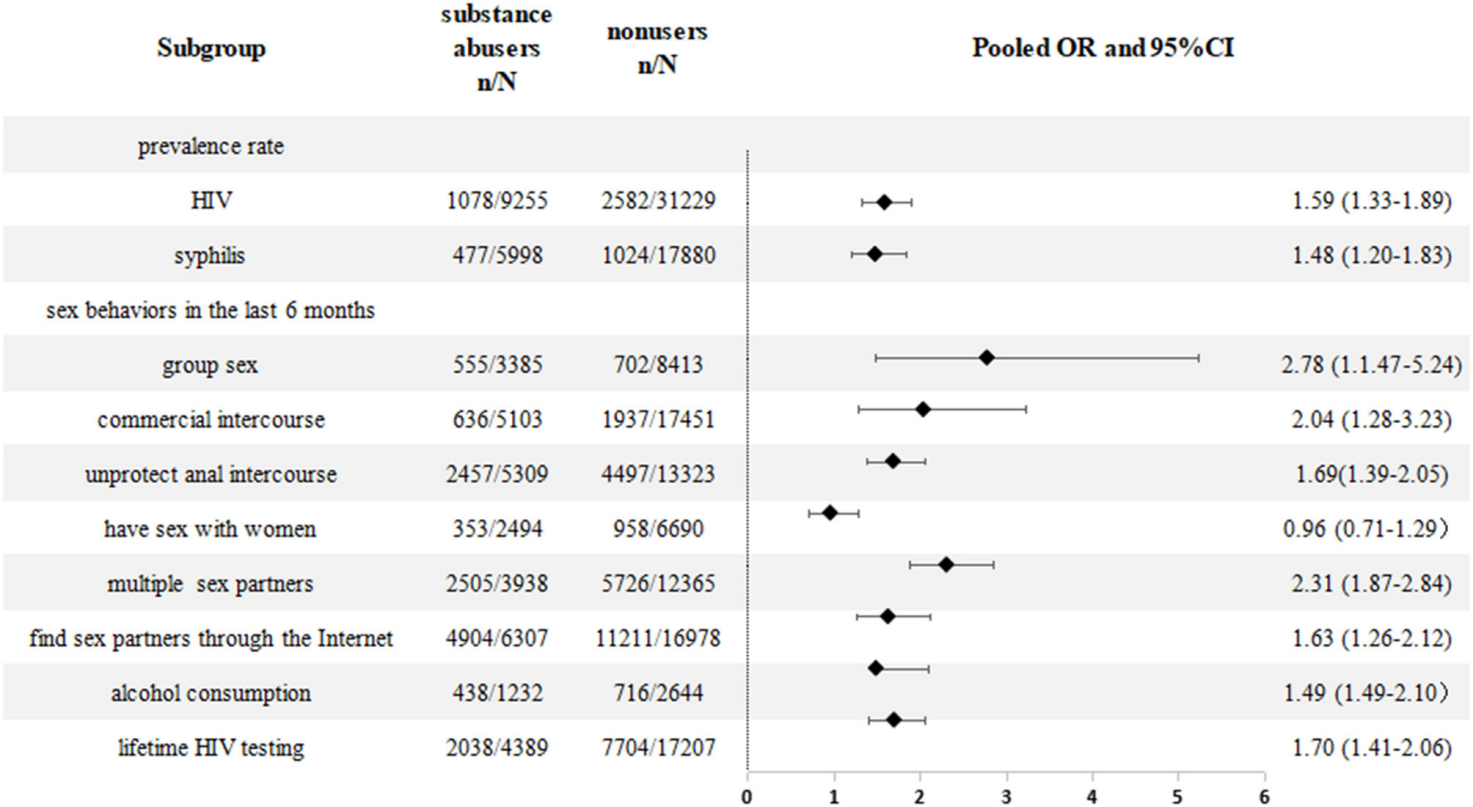
Meta-analyses comparison between substance abusers and non-users among Chinese MSM.

Subgroup analysis by location showed that the difference in pooled HIV prevalence was not significant (9.0% vs. 7.0%) between single-city and multi-cities; in the study period subgroup, the pooled prevalence rate of HIV among MSM from 2011 to 2018 was estimated to be 11% (95% CI: 0.08–0.13) and 7% (95% CI: 0.05–0. 09) after 2018; Subgroup analysis by sample size showed that the HIV prevalence for studies with sample size < 1,000, sample size of 1,000–2,000, and sample size >2,000 was: 8% (95% CI: 0.06–0.10), 9% (95% CI: 0.06–0.13), and 10.0% (95% CI: 0.08–0. 12), respectively. We also extracted information on syphilis infection status from 16 studies and found that the current prevalence of syphilis was higher among substance abusers than non-users (8.0% vs. 5.7%, pooled OR = 1.48 (95% CI: 1. 20–1.83); I^2^ = 67%, *P* < 0.01) (Figure 3).

### 3.5. Sexual risk behaviors

Forty-four studies compared sexual behaviors between substance abusers and non-users. Twenty studies reported that substance abusing MSM were more likely to find male partners through the internet, including WeChat and Blued (pooled percentages: 77.75% (4,904/6,307) vs. 66.03% (11,211/16,978); pooled OR = 1.63 (95% CI: 1.26–2.12); I^2^ = 91%; *P* < 0.01) (8, 18, 20, 24, 26, 28, 29, 34, 38, 47–49, 51, 53–56, 66, 67).

Sixteen studies found statistically significant for unprotected anal intercourse as substance abusers tended to have UAI in the last 6 months [pooled percentages: 46.28% (2,457/5,309) vs. 33.75% (4,497/13,323); pooled OR = 1.69 (95% CI: 1.39–2.09); I^2^ = 84%; *P* < 0.01] (8, 18, 20, 26, 28, 30, 34, 38, 47–49, 51, 53, 55, 66, 67). Further, MSM who reported ever using substance were 2.31 times more likely to have multiple sexual partners than non-users [pooled OR = 2.31 (95% CI: 1.87–2.84); I^2^ = 85%; *P* < 0.01] (18, 20, 25, 28–31, 47–49, 66, 67). Eleven studies included information on group sex between substance users and non-users. By comparison, MSM who reported ever using substance were 2.78 times more likely to engage in group sex in the past 6 months than non-users [pooled OR = 2.78 (95% CI: 1.47–5.24); I^2^ = 96%; *P* < 0.01] (8, 18, 20, 26, 28, 29, 42, 48, 55, 65, 67).

Seventeen studies found that substance abusers were more likely to have engaged in commercial sexual behavior in the past 6 months than non-users [pooled OR = 2.04 (95% CI: 1.28–3.23); I^2^ = 91%; *P* < 0.01] (5, 6, 8, 12, 15, 18, 26, 35, 37, 41–43, 47, 51, 65, 66). Also, substance abusers were more likely to report HIV testing in the past 6 months or a history of repeat HIV testing than non-users (pooled OR = 1.70, 95% CI: 1.41–2.06, I^2^ = 74%; *P* < 0.01) (8, 24, 27, 28, 30, 34, 39, 48, 51, 56, 66, 67). Five studies focused on alcohol consumption among MSM participants, and substance abusers were more likely to drink alcohol in the past 6 months [pooled OR = 1.49 (95% CI: 1.07–2.10; I^2^ = 70%; *P* < 0.01)] than non-users (6, 28, 31, 42, 51). Six studies have provided information about MSM who had sex with the women in the past 6 months [pooled OR = 0.96 (95% CI: 0.71–1.29); I^2^ = 75%; *P* < 0.01] (19, 34, 45, 48, 51, 65) (Table 4).

**Table 4:** Comparison of sexual behaviors between substance abusers and non-users among MSM in China.

**Influencing factors**	**Number of studies**	**Heterogeneity**		**Effect model**	**Pooled OR**	
		**I** ^2^ **/%**	***P*** **-value**		**OR**	**95% CI**
Syphilis infection	16	67	< 0.01	Random	1.48	1.20–1.83
HIV infection	28	88	< 0.01	Random	1.59	1.33–1.89
Unprotected anal sex	16	84	< 0.01	Random	1.69	1.39–2.05
Multiple sexual partners (≥2/1)	13	85	< 0.01	Random	2.31	1.87–2.84
Ways of finding sex partners (internet/others)	20	91	< 0.01	Random	1.63	1.26–2.12
Group sex in the past 6 months	11	96	< 0.01	Random	2.78	1.47–5.24
Commercial intercourse in the past 6 months	17	91	< 0.01	Random	2.04	1.28–3.23
HIV testing	12	74	< 0.01	Random	1.70	1.41–2.06
Having sex with women in the past 6 months	6	75	< 0.01	Random	0.96	0.71–1.29
Alcohol consumption	5	70	< 0.01	Random	1.49	1.07–2.10

### 3.6. Sensitivity analysis and publication bias

**Figure 4:**
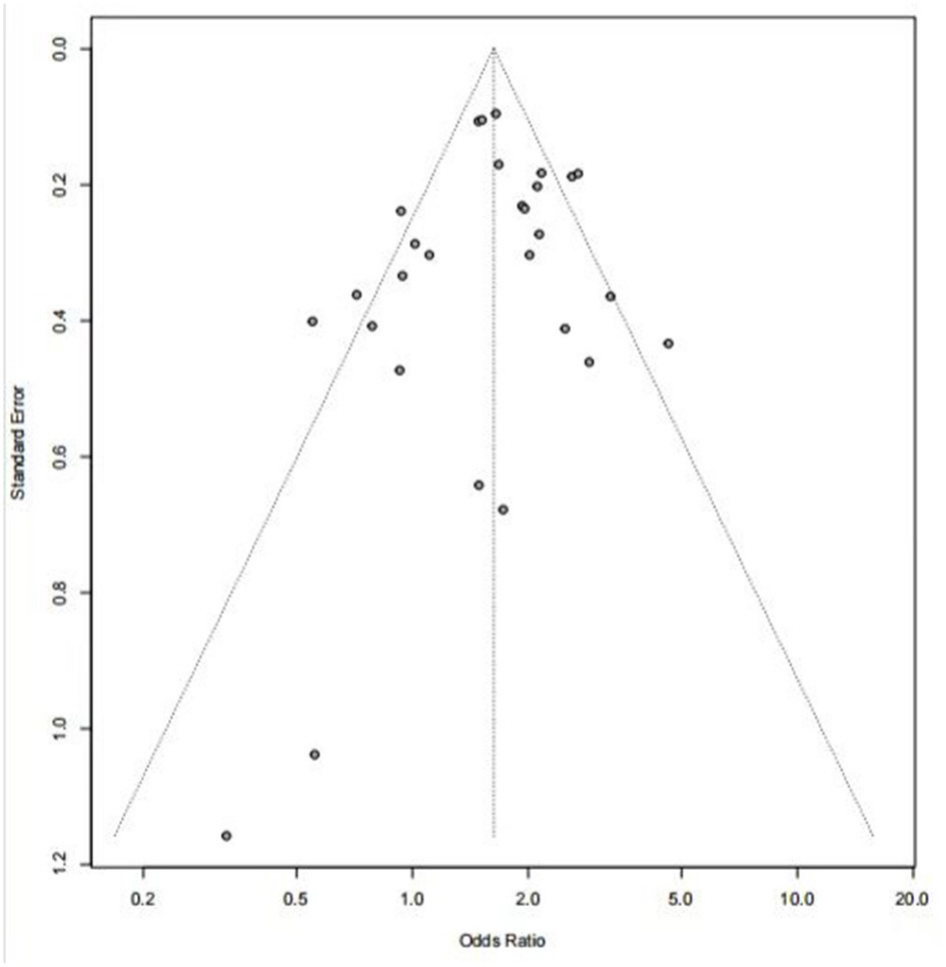
Funnel plot for assessing the publication bias of HIV prevalence between substance abusers and non-users.

None of the individual study's results affected the pooled estimate odds ratio. ORs remained similar in the sensitivity analysis, which suggested that the results were stable and reliable. Egger's test suggested no significant evidence of publication bias in comparing the prevalence of HIV (*P* = 0.51) between substance abusers and non-users (Figure 4).

## 4. Discussion

We conducted a quantitative review in China to assess the HIV/syphilis epidemic and the correlation between these epidemics and substance abuse and other sexual risk behaviors among MSM in China. This meta-analysis provides insight into HIV transmission among substance abusers. It also provides an important suggestion for China's public health sector to develop more effective prevention and intervention strategies for the MSM population. We integrated and analyzed data from 52 studies and further validated that the prevalence of HIV or syphilis among substance abusers of Chinese MSM is significantly higher than the general MSM population, similar to previous results (17, 22). We aggregated the characteristics, related sexual risk behaviors, and current HIV and syphilis status among Chinese MSM. We also identified the factors associated with HIV/syphilis infection between substance abusing MSM and non-users.

Our review showed a 23% (95% CI: 0.17–0.29) pooled proportion of Chinese MSM reported substance abuse, and in the past 6 months, a 25% (95% CI: 0.19–0.34) pooled proportion of Chinese MSM reported substance abuse. This finding is lower than the proportions reported among Chinese MSM in a global meta-analysis conducted in 2021 (*P* = 0.306, 95% CI: 0.238–0.373) (17). Furthermore, the pooled HIV prevalence was 10% among substance abusers, much higher than the HIV positivity rate in the general MSM population (*P* = 0.0507, 95% CI: 5.4%−6.1%) (2). The high HIV prevalence among substance-abusing Chinese MSM is a signal that, without effective intervention strategies, it may contribute to the HIV transmission risks among MSM will would lead to severe public health consequences. Our study results also showed that the substance abuse rate among Chinese MSM gradually increased over time. The proportion of substance-abusing MSM was about 19% (95% CI: 0.13–0.28) before 2018 and reached 27.0% (95%CI: 0.19–0.38) after 2018. One possible explanation for this phenomenon is that the rapid growth of the internet and the popularity of social media applications (such as Blued) have made MSM more susceptible to use substances. In recent years, a number of gay applications like Blued have been developed specifically for MSM social and sexual networking, increased used may facilitate seeking multiple sex partners (70, 71). Some applications targeting MSM have also emerged as important a venue through which MSM seek and maintain relationship with sex partners and substance abuse partners (72). The diversification of e-commerce platforms has provided convenient ways for MSM to obtain substances. It is worth noting that the rush popper and capsule zero were the most popular recreational drug among MSM. Although rush popper is banned or restricted in some countries like Canada and European Union (37), it is still not regulated as an illegal drug, and it can be easily obtained at a low cost online in China. In 2019, Chinese researchers investigated 2,616 MSM in six provinces through an online questionnaire (19). They found that 842 (32.2%) MSM had used at least one psychoactive substance, and 377 (14.4%) MSM reported using more than two psychoactive substances. For the MSM population, the rush poppers is still an important factor contributing to sexual behaviors, such as increasing sexual desire and reducing sexual inhibition (18). MSM were more likely to seek casual sex partners and engage in UAI after using the drug. Moreover, rush poppers are believed to prolong erection which prolongs anal intercourse, thus increasing the likelihood of anal bleeding and the risk of HIV infection (29, 65). It is strongly suggested that the public health sectors should pay attention to the issue of substance abuse, especially the rush poppers, and adopt targeted screening and detection for these subgroups of MSM.

Regarding the sexual risk behaviors among MSM, our study suggests that Chinese substance abusers were more likely to seek sex partners through the internet or gay applications than non-users in the last 6 months. These social media applications greatly facilitate the identification of sexual partners and do not have any restrictions on the time or location (70, 71). In addition, these applications also provide a platform for individuals to organize private parties where the combination of drug use and gay applications may contribute to an increase in the risk of HIV transmission among MSM (24).

Consistent with previous studies, our study suggests that substance abuse is associated with unprotected anal intercourse, commercial sex, and group sex (73–75). Similar results were reported by a United States study which found that substance-abusing MSM were twice more likely to have had commercial sex in the last 6 months than non-users (9). This result was lower than the 5.07 odds found in the study by Robert et al. abroad (76). The high level of sexual activity among substance abusers might make it more difficult to maintain a stable and regular sexual partner relationship. Drug abuse was positively associated with multiple sex partners during a sexual encounter (77, 78), and unprotected sex encounter is more likely to occur during participation in group sex. Moreover, it has been demonstrated that the perianal skin is more susceptible to damage during unprotected anal intercourse and therefore provides optimal conditions for the transmission of HIV.

Alcohol consumption among MSM may also be a cause for concern although only five included papers provided information to compare substance abusers with non-users (6, 45, 51, 62, 73). Substance users were more likely to drink alcohol, and the combination of alcohol and drug use may reduce the sense of restraint and increase the sexual risk behaviors (15, 49, 51).

In our study, pooled HIV testing prevalence was higher among substance abusers than non-users. Substance abusers seem more likely to engage in high-risk sex behaviors (such as group sex, commercial sex, and unprotected anal intercourse) than non-users. Thus, they are more likely to use preventative methods, including frequent HIV and STI testing. The HIV and syphilis prevalence rates were higher among substance abusers than non-users, which suggested that MSM with a history of substance abuse were particularly more likely to develop HIV/syphilis infection. Compromised immunity due to syphilis infection may increase the risk of HIV acquisition and facilitate HIV transmission. Hence, screening and referral efforts for sexually transmissible infections are needed.

This meta-analysis is not without several limitations. First, as the cross-sectional surveys pooled together, it is difficult to infer the temporal sequence between substance abuse and HIV/Syphilis infection. Second, as respondents' drug abuse has not been confirmed by standardized laboratory testing, reporting bias and recall bias could affect the veracity of the results and thus underestimate the HIV prevalence of MSM. Third, some studies included in this meta-analysis used convenient samples or a cross-sectional design. The resulting selection bias may have affected the veracity of our results. Moreover, for a lack of surveys from rural areas, we could not further explore the impact of substance abuse on the infection of HIV/Syphilis among all Chinese MSM.

## 5. Conclusion

This study highlights the correlation between substance abuse and HIV/Syphilis infection. Substance abuse among the MSM population can increase the rate of high-risk sexual behaviors and facilitate HIV/Syphilis infection. The mechanism still needs to be explored and strengthened. Targeted knowledge promotion and expanding diagnostics interventions among high at-risk populations should be adopted for HIV/Syphilis prevention. Meanwhile, care providers should pay attention to substance abusers and related high-risk behaviors and encourage safe sex practices. Moreover, intervention programmers should utilize internet-based social organizations to provide peer education and implement social discrimination and HIV-related stigma reduction interventions to help slow the spread of HIV/Syphilis.

## Data availability statement

The original contributions presented in the study are included in the article/Supplementary material, further inquiries can be directed to the corresponding authors.

## Author contributions

HL, XG, and GF contributed to the manuscript's conception, design, and review. GC and TZ carried out the data collection and drafted the first manuscript. TZ and CS performed the statistical analysis. All authors contributed to the manuscript revision and approved the submitted version.
