# Supplementary material for: The epidemic of HIV and syphilis and the correlation with substance abuse among men who have sex with men in China: A systematic review and meta-analysis
Source: Front Public Health. 2023 Feb 17;11:1082637. doi: 10.3389/fpubh.2023.1082637 (PMC9982104; doi:10.3389/fpubh.2023.1082637)
Supplement: Supplementary file 1 [file Data_Sheet_1.docx]

| **Summary of quality assessment score of 52 studies** | | | | | | | |
| --- | --- | --- | --- | --- | --- | --- | --- |
| **Authors** | **Publication year** | **Q1** | Q2 | Q3 | Q4 | Q5 | **QATSO Scores** |
| Gang Liu | 2010 | 0 | NA | 1 | 1 | 1 | 3 |
| Shengjun Xi | 2011 | 0 | 1 | 1 | 1 | 1 | 4 |
| Yifei Hu | 2014 | 0 | 1 | 1 | 1 | 1 | 4 |
| Dongliang Li | 2014 | 0 | 1 | 1 | 1 | 1 | 4 |
| Wanping He | 2014 | 0 | 1 | 1 | 1 | 1 | 4 |
| Li Liu | 2015 | 0 | 1 | 1 | 1 | 1 | 4 |
| Zixin Wang | 2015 | 0 | NA | 1 | 1 | 1 | 3 |
| Xi Chen | 2015 | 0 | 1 | 1 | 1 | 1 | 4 |
| Yumao Cai | 2016 | 0 | 1 | 1 | 1 | 1 | 4 |
| Hui Shi | 2016 | 0 | 1 | 1 | 1 | 1 | 4 |
| Yumiao Wang | 2016 | 0 | 1 | 1 | 1 | 1 | 4 |
| Mengqing Chen | 2016 | 0 | 0 | 1 | 1 | 1 | 3 |
| Dongliang Li | 2016 | 0 | 1 | 1 | 1 | 1 | 4 |
| G.L. Yang | 2016 | 0 | 0 | 1 | 1 | 1 | 3 |
| zhang chen | 2016 | 0 | 1 | 1 | 1 | 1 | 4 |
| Hehong Jiang | 2017 | 0 | 1 | 1 | 1 | 1 | 4 |
| Zhengping Zhu | 2017 | 0 | 1 | 1 | 1 | 1 | 4 |
| Yuanyuan Xu | 2017 | 0 | 1 | 1 | 1 | 1 | 4 |
| Xiaohua Yang | 2017 | 0 | NA | 1 | 1 | 1 | 3 |
| Hua Jia | 2017 | 0 | 1 | 1 | 1 | 1 | 4 |
| Zheng Zhang | 2017 | 0 | 1 | 1 | 1 | 1 | 4 |
| Xiaofang Wang | 2017 | 0 | 1 | 1 | 1 | 1 | 4 |
| Duo Shan | 2017 | 0 | 1 | 1 | 1 | 1 | 4 |
| **Summary of quality assessment score of 52 studies(continued)** | | | | | | | |
| **Authors** | **Publication year** | **Q1** | Q2 | Q3 | Q4 | Q5 | **QATSO Scores** |
| Chenlin Duan | 2017 | 0 | 1 | 1 | 1 | 1 | 4 |
| Peizhen Zhao | 2017 | 0 | 0 | 1 | 1 | 1 | 3 |
| Na Han | 2018 | 0 | 1 | 1 | 1 | 1 | 4 |
| Shan Lu | 2018 | 0 | 1 | 1 | 1 | 1 | 4 |
| Xiaoyi Zhou | 2018 | 0 | 1 | 1 | 1 | 1 | 4 |
| He Lin | 2018 | 0 | 1 | 1 | 1 | 1 | 4 |
| Lan Guanghua | 2018 | 0 | 1 | 1 | 1 | 0 | 3 |
| Meng Li | 2018 | 0 | 1 | 1 | 1 | 1 | 4 |
| Dechuan Zhang | 2019 | 0 | NA | 1 | 1 | 1 | 3 |
| Qing Duan | 2019 | 0 | 1 | 1 | 1 | 1 | 4 |
| Yuanyuan Xu | 2019 | 0 | 1 | 1 | 1 | 1 | 4 |
| Yingxue Dai | 2019 | 0 | 1 | 1 | 1 | 1 | 4 |
| Feng Zhou | 2020 | 0 | 1 | 1 | 1 | 1 | 4 |
| Zhenyu Wang | 2020 | 0 | 1 | 1 | 1 | 0 | 3 |
| Yao Li | 2020 | 0 | 1 | 1 | 1 | 1 | 4 |
| Pengxiang Huang | 2020 | 0 | 1 | 1 | 1 | 1 | 4 |
| Zhikan Ni | 2020 | 0 | 1 | 1 | 1 | 1 | 4 |
| Chen Jin | 2020 | 0 | 1 | 1 | 1 | 1 | 4 |
| Xueer Guo | 2020 | 0 | 0 | 1 | 1 | 1 | 3 |
| Yao Yan | 2020 | 0 | NA | 1 | 1 | 1 | 3 |
| Li Li | 2021 | 0 | NA | 1 | 1 | 1 | 3 |
| Xiang Mao | 2021 | 0 | 1 | 1 | 1 | 1 | 4 |
| Duo Shan | 2021 | 0 | 1 | 1 | 1 | 1 | 4 |
| Zeyang Yu | 2021 | 0 | 1 | 1 | 1 | 1 | 4 |
| Lixuan Wang | 2022 | 0 | 1 | 1 | 1 | 1 | 4 |
| Jianzhuo Li | 2022 | 0 | 1 | 1 | 1 | 1 | 4 |
| Zhenxing Chu | 2013 | 0 | 1 | 1 | 1 | 1 | 4 |
| Huanhuan Li | 2019 | 0 | 1 | 1 | 1 | 1 | 4 |
| Erlei Peng | 2019 | 0 | 1 | 1 | 1 | 1 | 4 |
